# The Book World of Medicine and Science

**Published:** 1903-04-25

**Authors:** 


### THE BOOK WORLD OF MEDICINE AND SCIENCE.

Practical Details of Cataract Extraction. By H. Herbert, F.R.C.S., Eng., Major, I.M.S., Professor of Ophthalmic Medicine and Surgery, Grant Medical College, etc. 12mo. pp. 109. (Baillière, Tindall and Cox. 1903.)

Cataract is, we believe, very common and very widely diffused throughout India; and Major Herbert's little book would possibly be helpful to any practitioner who found himself among a population containing a large proportion of sufferers, and who wished to do his best for them, but who, as is only too common, had during his studentship regarded ophthalmic work as something which would not be likely to "pay" before a board of examiners. It gives a concise account of the methods of operating under different conditions, and a certain amount of guidance concerning diagnosis. It is of necessity superficial, but, as far as we have seen, it is nowhere incorrect. The position of the author has given him large practical experience; but his sound teaching has nowhere deviated into originality.

Disciplinary Civic and Moral Education. By Llewellyn Wynn Williams, B.Sc., Honorary Secretary, Society for the Reform of School Discipline. (London: Simpkin, Marshall, Hamilton, Kent and Co., Ltd. 1903. Price 2s. 6d. net.)

Mr. Williams writes, as may be inferred from his title-page, as one of the opponents of corporal punishment, and he certainly makes out a strong case against it. Corporal punishment, when inflicted on a child for failure to understand or remember a lesson is futile, or worse, in that, if it has any effect at all, the physical pain which he is suffering must unfit the child for attending to anything of an intellectual nature. When the punishment is inflicted for moral offences, it certainly makes clear to the child that these offences are liable to be found out, and that therefore it does not pay to commit them—unless with better safeguards against discovery. But this can hardly be called moral teaching. Mr. Williams ignores, however, what is the practical reason why corporal punishment is permitted in schools. It is that the fear of pain may make a wild or restless child forbear from distracting the attention of a large number of quiet pupils, and thus rendering the instruction they are receiving of no effect. A single teacher may hold the attention of a class of from 20 to 25 pupils, but only one in a thousand can keep a real grip of a class of sixty or more, such as is frequently found in our elementary schools. This, however, we admit is more properly an indictment of large classes than a defence of corporal punishment. And there can be no doubt that the power of inflicting corporal punishment entrusted to teachers has often been much abused. It is safe to say that no assistant teacher should be empowered to thrash a child. Where this power is stopped, there is a great diminution in the number of whippings performed in a school. A teacher hesitates about reporting a pupil to the headmaster for punishment, unless for some serious fault, knowing well that to send up children frequently implies a lack of power on his or her own part to maintain discipline. In cases where the fault is a failure to understand or remember a lesson, the obvious punishment is a detention until the lesson is learnt. This, of course, would involve the teacher staying in also, and is perhaps for this reason less popular than the flogging. Mr. Williams speaks in high praise of the "School City" system, which is popular in the United States, by which the discipline of the school is put largely into the hands of the children themselves, who are thus encouraged to take a pride in preserving order and keeping up the honour of the school. A plan by which children judge their fellows, and inflict punishments—of course not corporal—in cases of fault, seems to our minds liable to abuse, for school popularity is not always regulated by moral considerations, and many a mischievous urchin might escape if he were liked by his schoolfellows. But those who have tried the plan say that not only does the putting the discipline of the school in the hands of the pupils tend to prevent breaches of it, but the sense of personal responsibility and citizenship helps to make the child judges and other functionaries careful and just in their verdicts. Mr. Williams' book is full of thought and interest, and whether one ageees with him on every point or not, one cannot but feel that the suggestions he makes are worth considering. Every one interested in our educational system should peruse this book.

Massage and the Original Swedish Movements: Their Application to Various Diseases of the Body. By Kurre W. Ostrom. Fifth Edition. Revised and enlarged with 115 illustrations. (London: H. K. Lewis, 136 Gower Street, W.C. 3s. 6d. net.)

This is an exhaustive treatise on Massage and kindred movements, is most abundantly and lucidly illustrated, and treats of the subject from the earliest ages when it was practised to our own times. Students of the art—and it is useless, if not dangerous, unless seriously studied—will find this a comprehensive text-book, and, moreover, the author has added a list of forty-four works on the same subject by writers of various nationalities, which adds greatly to the value of the book. The "General Remarks" and the chapter on "The Massage Treatment in America" are very important and interesting, especially in regard to the necessity of proper registration of trained masseurs, for their own and the public's protection. The Swedish movements, the Schott treatment, and massage in special diseases are separately and methodically described after the chapters on General Massage. The great value of massage applied to stiff joints after fractures and bullet-wounds is perhaps worthy of more insistence (p. 163).. In the late South African war the importance of this mode of treatment was clearly demonstrated, and to at least one big base hospital (*e.g.*, the Imperial Yeomanry Hospital, Deelfontein, Cape Colony) a professional masseur was attached, who did a vast amount of good work by restoring stiff elbows and other wounded joints to their original mobility. Enough has, however, been said to recommend Mr. Ostrom's book to all earnest students of the therapeutic art of massage.

Cancer: Theories of the Parasitic Origin. By W. Roger Williams, F.R.C.S. Reprint from the Supplement of The Twentieth Century Practice of Medicine. Vol. XXI. 1903.

Mr. Roger Williams' monograph affords an admirable summary and criticism of the various theories and observations which have sought to throw light on the different views regarding the parasitic origin of cancer. Although presenting no new evidence it supplies a convenient compilation of much scattered, and in some instances not readily accessible, work, and should prove of considerable service to serious students. But in the reprint before us there are no references to original papers, which is a serious omission, and one which greatly lessens the permanent value of the monograph.

